# Effects of Herring Milt Hydrolysates and Fractions in a Diet-Induced Obesity Model

**DOI:** 10.3390/foods10092046

**Published:** 2021-08-31

**Authors:** Noémie Benoit, Marie-Julie Dubois, Geneviève Pilon, Thibault V. Varin, André Marette, Laurent Bazinet

**Affiliations:** 1 Laboratory of Food Processing and Electromembrane Process (LTAPEM), Department of Food Sciences, Université Laval, Québec, QC G1V 0A6, Canada; noemie.benoit.1@ulaval.ca; 2 Institute of Nutrition and Functional Foods (INAF), Université Laval, Québec, QC G1V0A6, Canada; marie-julie.dubois@criucpq.ulaval.ca (M.-J.D.); genevieve.pilon@criucpq.ulaval.ca (G.P.); thibaut.varin.1@ulaval.ca (T.V.V.); andre.marette@criucpq.ulaval.ca (A.M.); 3 Department of Medicine, Québec Heart and Lung Institute, Université Laval, Québec, QC G1V 4G5, Canada

**Keywords:** herring milt hydrolysate, bioactive peptides, obesity, glucose tolerance, microbiota

## Abstract

Over the past years, promising results from studies have shown that herring milt hydrolysates (HMH) can counter immune-metabolic disorders associated with obesity. However, more studies must corroborate these results. Thus, three commercial hydrolysates (HMH1, HMH2, and HMH3) as well as the fractions of two of them (HMH4 and HMH5) obtained by electrodialysis with ultrafiltration membranes (EDUF) were evaluated *in vivo* at higher doses compared to a previous study. To achieve this, seven groups of mice were fed for 8 weeks with either a control Chow diet or an obesogenic diet rich in fat and sucrose (HFHS) and supplemented by daily gavage with water or 312.5 mg/kg of one of the five HMH products. In summary, HMH supplements had no impact on weight gain. In the insulin tolerance test (ITT), HMH2 and its HMH5 fraction significantly reduced the blood sugar variation (*p* < 0.05). However, during the glucose tolerance (OGTT), HMH2 supplement increased the hyperinsulinemia variation (*p* < 0.05) induced by the HFHS diet. HMH1, HMH2, and HMH5 supplements generated potentially beneficial changes for health in the gut microbiota. These results reveal that HMH do not counteract obesity effects but may decrease certain physiological effects induced by obesity.

## 1. Introduction

Metabolic syndrome is often referred to as the disease of the century, since one in five Canadian adults suffers from it [1]. This is a health disorder that can group together several risk factors (fasting hyperglycemia, high blood pressure and high triglyceride levels, obesity and low high-density lipoprotein (HDL) cholesterol levels) in the same individual [2]. These risk factors increase the propensity to develop chronic diseases. In Canada, the risk of dying from these diseases has decreased since the end of the 1990s [3], however they were still responsible for 88% of deaths in 2015 [4]. The number of people with one or more chronic diseases is also constantly increasing, which has an economic impact [3]. In Canada, two-thirds of health care costs are attributable to these chronic societal diseases [5]. The solution proposed by the World Health Organization (WHO) [6] lies in the prevention of these diseases by controlling risk factors. Since these risks stem in large part from poor lifestyle habits, a healthy lifestyle including, for example, a good diet should be advocated [6].

Over the past decades, several studies have shown the positive impact of seafood products consumption on health [7,8,9]. These health benefits have often been explained by the high concentration in polyunsaturated fatty acids, such as omega-3, in fatty fish. Moreover, the peptides contained in these foods also play an important role. However, to access these peptides, a hydrolysis step needs to be performed on the fish or marine by-product. Following the protein hydrolysis, the peptides obtained are then available to play their bioactive roles since they can display numerous bioactivities. For example, *in vivo*, salmon peptides would prevent glucose intolerance, inflammation, and dyslipidemia [10], while Atlantic salmon, halibut, and tilapia by-product peptides would influence glucose tolerance [11,12]. At the same time, several studies have focused on the valorization of marine by-products, such as Alaskan pollock by-products whose peptides would have antioxidant capacity [13,14] and snow crab by-product hydrolysates whose peptides would have anticancer properties [15]. More recently, Durand et al. [16] were interested in hydrolysates from herring milt. *In vivo*, two of these commercial hydrolysates rapidly improved oral glucose tolerance [17], and, after electrodialysis with ultrafiltration membrane (EDUF) separation process at laboratory scale, one of these hydrolysates has shown *in vitro* an impact on improving glucose uptake [16]. The electrodialysis experiments carried out to obtain the different fractions therefore make it possible to increase the bioactivity of a product. The objective of this work was to validate *in vivo* the effect of three industrial products and two fractions resulting from their EDUF separation at semi-industrial scale, provided as a supplement to a diet rich in fat and sugar in mice, on associated physiological functions in the development of metabolic disorders combined with obesity. This will help to establish whether the different supplements offer favorable modulation towards certain physiological factors, including insulin sensitivity, glucose tolerance, as well as the composition and diversity of the gut microbiota.

## 2. Materials and Methods

### 2.1. Supplementation

**Figure 1:**
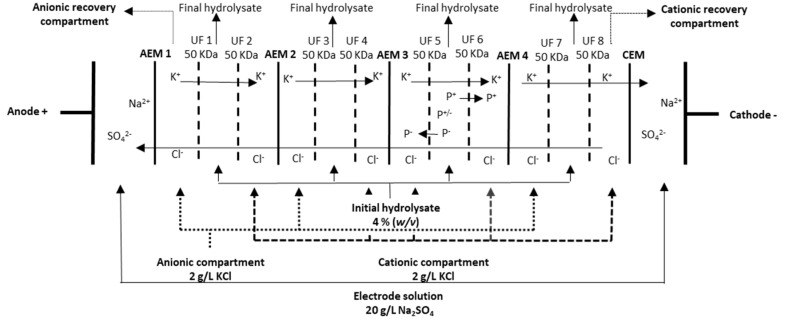
EDUF configuration used for the separation and production of HMH4 and HMH5; P^+/−^ neutral peptides, P^+^ cationic peptides, P^−^ anionic peptides [19].

Five products based on herring milt hydrolysates (HMH1, HMH2, HMH3, HMH4, HMH5) were studied as supplementation of the high-fat, high-sucrose (HFHS) diet. Their chemical composition is given in Table 1. Three of them (HMH1, HMH2, HMH3) are the industrial products provided by Ocean NutraSciences (Matane, QC, Canada). The company employs a confidential mix of enzyme to perform the hydrolysis and, afterwards, separates the hydrolysates obtained by different filtration steps. The permeate formed the HMH1, the retentate formed the HMH2, while the HMH3 was a mix of the retentate and astaxanthin. The other supplementations (HMH4, HMH5) were obtained following the separation of HMH1 and HMH2 by EDUF (pH 7 during 6 h, constant voltage) (Figure 1). They were afterwards demineralized by conventional electrodialysis (ED) based on the work of Durand et al. [16,18]. Finally, HMH4 is the peptide fraction collected in the cationic recovery compartment resulting from the EDUF separation of HMH1, whereas HMH5 is the final hydrolysate following EDUF separation of HMH2. The choices of fractionation and resulting compartments used for testing were based on the previous results from Durand et al. [16,18], who demonstrated *in vitro* bioactivities of these fractions. Each product was given daily through oral gavage at a dose of 312.5 mg/kg, which is an equivalent of 1.5 g/day in humans (60 kg). The products were dissolved in animal facility water that had previously been demineralized by reverse osmosis, remineralized using a softener, filtered, UV treated, and chlorinated at 2.5 ppm. The solution was made up to 39 mg/mL and aliquoted then stored at −20°C. Each morning, an aliquot was thawed, and the solutions were administered according to the weight of the animal (200 µL/25 g) at a dose of 312.5 mg/kg.

### 2.2. Animals and Dietary Treatment

C57Bl/6j male mice were received from the Jackson Laboratory at 6 weeks of age. They were individually housed in ventilated cages, in a controlled environment (12 h daylight cycle), and fed with a Chow diet (Teklad diet 2018) *ad libitum* for 12 days. After this acclimatization period, mice were segregated in 7 groups (*n* = 12) and randomly attributed one of the treatments as follows: Chow control force-fed daily with water (Chow, 3.1 kcal/g), HFHS diet providing 65% of energy from fat, force-fed daily with water (HFHS, 5.49 kcal/g), and a diet HFHS supplemented with one of the different products HMH1, HMH2, HMH3, HMH4, and HMH5 for the last five groups. Obesity was determined to be diet-induced, since this type of model best simulates aspects of the metabolic syndrome observed in humans [20]. The HFHS diet, often used in *in vivo* studies to induce obesity [21,22,23], was specifically selected to keep the same parameters as in the study by Durand et al. [17]. Considering the daily dose and the values presented in Table 1, the amount of energy potentially provided by the force-feeding solution was negligible and, therefore, was not considered. Food intake was measured three times a week, and body weight was assessed twice a week. Before treatment (T0) and at week 8 (T8), body composition was assessed by nuclear magnetic resonance using Bruker’s Minispec LF90II (Bruker Optics, Milton, ON, Canada). Following the 8 weeks of treatment, mice were euthanized by cardiac puncture under isoflurane anesthesia. Tissues and organs were collected, quickly frozen in liquid nitrogen, and stored at −80 °C until further experiments. This protocol study (no. 2019062-1) was approved by the Laval University Animal Ethics Committee in addition to meet the Guidelines for the care and use of laboratory animals.

### 2.3. Insulin Tolerance Test

An Insulin Tolerance Test (ITT) was conducted at week 6 after a 6 h fast. Glycemia was measured using a One Touch Verio Flex glucometer (LifeScan) before (0 min) and after (10, 20, 30, and 60 min) intraperitoneal insulin injection (0.65 U/kg), in blood from tail vein.

### 2.4. Glucose Tolerance Test

Three days before the sacrifice, at week 8, the oral Glucose Tolerance Test (OGTT) was realized on mice after an overnight 12 h fast. Each mouse received, by force-feeding, a solution of dextrose at 1 g/kg of body weight. Blood samples (60 μL) were collected by tail vein at times 0, 15, 30, 60, and 120 min, to measure glycemia and insulinemia.

### 2.5. Insulin Measurement

Plasma insulin was measured using a mouse ultrasensitive insulin ELISA kit according to the manufacturer’s instructions (Alpco, Salem, MA, USA).

### 2.6. Gut Microbiota Analysis

Fresh fecal samples were gathered at weeks 0 and 8 then frozen at −80 °C until further analysis. DNA was extracted using the NucleoSpin^®^ 96 Soil kit (Macherey-Nagel, Bethlehem, PA, USA) following the manufacturer’s instructions. Quant-iT ™ PicoGreen^®^ dsDNA assay kit (Invitrogen, Eugene, OR, USA) was used to measure DNA concentration and samples were sent to the Centre d’expertise et de services Génome Québec (Montréal, Canada) for sequencing. 16S RNA gene-based profiling of fecal microbiota was performed according to Choi et al. [24]. To normalize sampling effort, samples were rarefied to an even sampling depth of 20.951 sequences. To quantify bacterial alpha-diversity, Shannon index was calculated. Beta diversity was assessed by principal component analysis (PCA) based on Aitchison distance calculated from unrarefied data. Linear discriminant analysis effect size (LEfSe) was performed to identify genera differentially enriched in the between-group comparisons. A *p*-value of <0.05 and a linear discriminant analysis (LDA) score ≥2.5 were considered statistically significant.

### 2.7. Statistical Analyses

All statistical analysis of *in vivo* results were performed using Graphpad prism software (Version 8.0, Graphpad Software Inc., San Diego, CA, USA). Data from the HFHS group were compared to those from the Chow group by a Student’s t test to analyze the effects of this diet. Data from diets supplemented with HMH were then evaluated by Dunnett’s test followed by one-way ANOVA. When analyzing repeated measures over time, such as for food intake, ITT, and OGTT, a two-way ANOVA were applied [25]. 

## 3. Results and Discussion

### 3.1. Physiological Effects

Physiological data displays the effects of the HFHS diet compared to Chow diet as well as supplementation with HMH in comparison with the HFHS diet (Table 2). Firstly, all mice on the HFHS diet (supplemented or not with HMHs) had significant (*p* < 0.001) weight gain compared to the Chow group. The HFHS diet also significantly increased total energy intake (+20.98%, *p* < 0.001), visceral fat pad (+193.51%, *p* < 0.001), subcutaneous fat pad (+124.65%, *p* < 0.001), brown adipose tissue (+45.21%, *p* < 0.01), and total fat mass (+168.38%, *p* < 0.001). However, no impact of the HFHS diet nor HMH supplementation was noticed on the total lean mass and the weight of the different organs recovered, except for the brain, which was statistically larger (*p* < 0.05) in the mice from HMH1 group compared to the HFHS group.

The physiological results allowed to conclude that the HMH supplementation did not limit or worsen the development of obesity caused by the HFHS diet. The previous results obtained by Durand et al. [17] were similar to those of the present study with the exception of the HMH2 supplementation, which led to larger weight gain than what Durand et al. [17] observed (7.83 ± 0.60 g). Such comparison between the two studies confirms that increasing the daily dose of supplement from 208.8 mg/kg [17] to 312.5 mg/kg (present study) did not minimize the effects of the HFHS diet physiological parameters or increase the development of obesity [17]. Finally, the HMH1 supplementation increased the brain weight, which was not observed in previous studies by Durand et al. [17]. In-depth analysis on the brain will be necessary to understand the underlying mechanisms behind this observation and to determine if this increase in brain weight was positive or negative on the animal’s health.

### 3.2. Insulin Tolerance

**Figure 2:**
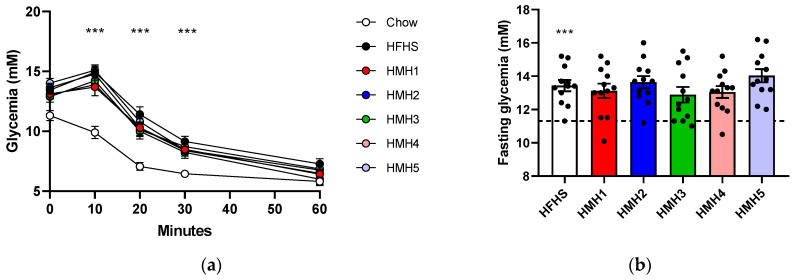
Impact of HMHs’ supplementation in mice fed with HFHS on insulin tolerance during the ITT (**a**) Glycemic curve as a function of time after administration of insulin (**b**) Blood glucose concentration was measured at t = 0 after 6 h of fasting. *** *p* ˂ 0.001 Chow vs. HFHS.

ITT was performed at week 6 and the results are shown in Figure 2. Ten minutes after insulin injection the glycemia of the HFHS groups started to decrease to ultimately reach a close or similar level to the Chow group after 60 min. During the first 30 min of the test, mice fed with HFHS diet presented a glycemia level statistically higher (*p* < 0.001) than mice of the Chow group (Figure 2a). Fasting hyperglycemia in HFHS groups was also observed (Figure 2b).

The ITT was an indication of whether the mice had a normal glycemic reaction when faced with an insulin injection. The statistically higher glycemia of the HFHS groups during the first 30 min confirmed the expectation that the HFHS diet increased the basal glycemia and generated a certain level of insulin resistance, but the HMHs’ supplementation at the dose given here did not reverse this trend. This was also observed by Durand et al. [17] for a lower dose (208.8 mg/kg).

**Figure 3:**
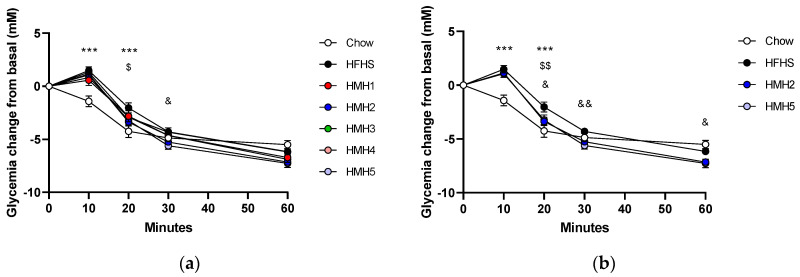
Impact of HMH supplementation in mice fed with HFHS on insulin tolerance during the ITT. Glycemic variation curve as a function of time (**a**) to statistically compare the different supplementations with HFHS (**b**) focus more specifically on the statistics of HMH2 and the resulting product HMH5. *** *p* < 0.001 Chow vs. HFHS, $ *p* < 0.05 HFHS vs. HMH2, $$ *p* < 0.01 HFHS vs. HMH2, & *p* < 0.05 HFHS vs. HMH5, && *p* < 0.05 HFHS vs. HMH5.

When results were expressed as a variation of glycemia from basal (Figure 3a) it appeared that HMH2 and HMH5 supplementations improved the response to insulin at 20 and 30 min, respectively. In addition, Figure 3b clearly displays that HMH5 glycemia variation was statistically lower at times 20, 30, and 60 min when only the fraction and its original product (HMH2) were considered in the comparison with HFHS diet. Although these results were not clearly upheld in previous studies, Durand et al. [16,17] had shown an improvement in glycemia by HMH2 during OGTT and a capacity of HMH5 to improve glucose uptake *in vitro*. The polyunsaturated fatty acids (PUFAs) that were present in HMH2, as described in previous studies, were possibly still found in the HMH5 fraction because this was the final fraction of HMH2 obtained after EDUF, and this process mainly targets the migration of peptides and charged ions [16]. According to some studies, PUFAs reduce insulin resistance and, therefore, affect the glycemic response [26,27]. For example, Taouis et al. [27] explained that supplementation with PUFAs given to an HFHS diet would restore *in vivo* the phosphorylation of IRS-1 tyrosine (tyrosine phosphorylation of insulin receptor substrate) in the muscle and allow proper functioning of PI-3K and GLUT4, which play a role in the regulation of glucose by insulin.

### 3.3. Glucose Tolerance

**Figure 4:**
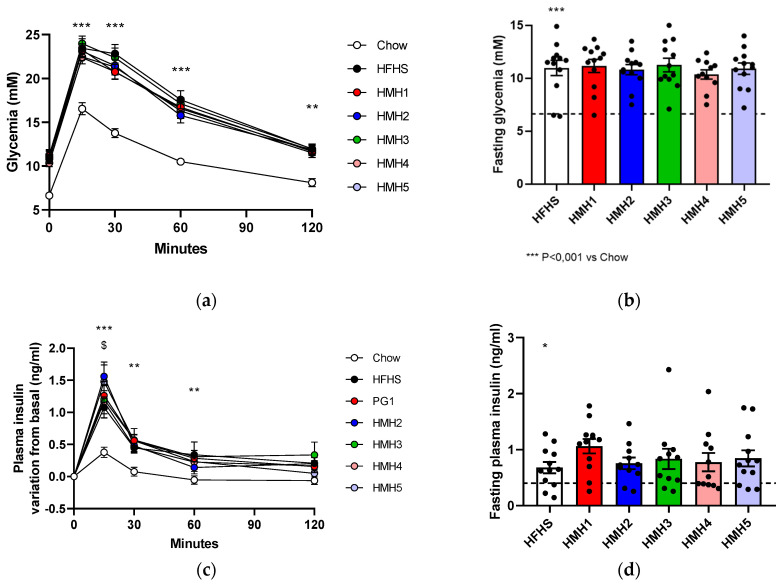
Impact of HMHs’ supplementation in mice fed with HFHS on glucose tolerance during OGTT. (**a**) Glycemia curve as a function of time after administration of dextrose (1 g/kg of body weight), (**b**) Blood glucose concentrations were measured at t = 0 after 12 h of fasting, (**c**) Insulin variation curve product as a function of time, and (**d**) Insulin concentrations were measured at t = 0 after 12 h of fasting. * *p* < 0.05 Chow vs. HFHS, ** *p* < 0.01 Chow vs. HFHS, *** *p* < 0.001 Chow vs. HFHS, $ *p* < 0.05 HFHS vs. HMH2.

The OGTT was performed at week 8. Glycemia and, thus, blood glucose concentrations in HFHS mice were statistically higher (*p* < 0.001) than in Chow animals in the fasting state and throughout OGTT (Figure 4a,b). The OGTT was used to assess insulin production in response to an increase of glycemia (Figure 4c). The increase in insulin variation for HMH2 at 15 min was statistically higher (*p* < 0.05) than for HFHS. Furthermore, the amount of insulin produced that was statistically higher in the fasting state for the HFHS groups (Figure 4d) remained statistically (*p* < 0.05) higher throughout the 120 min despite large variations (results not shown).

As expected, the significant difference between the Chow diet and the HFHS groups suggested an onset of glucose intolerance. In contrast to the previous study by Durand et al. [17], where the HMH2 and HMH3 supplementations displayed a reduction in glycemia compared to the HFHS diet (t = 15 min), in the present study, no HMHs’ supplementation had a significant impact on the HFHS diet-induced glucose intolerance. However, the comparison of the AUCs (Aera Under the Curve) (results not shown) for both studies displayed no differences between the mice of HFHS groups, suggesting no effect of HMH2 and HMH3 supplementations on glucose intolerance induced by HFHS diet [17]. Also, the glucose intolerance observed above in the groups fed with HFHS would therefore have led to a slight hyperinsulinemia. In the long term, such deregulations could cause problems with the production of insulin by the pancreas as with type 2 diabetes, but here it does not appear to be the case. The HMHs’ supplementation did not impact the insulinemia, except for HMH2, which worsened the hyperinsulinemia caused by HFHS, which shows that the different groups who received the HFHS diet are not diabetic. The specific comparison of HMH2 and HMH5 with HFHS and Chow diets (without HMH1, HMH3, and HMH4 groups) (results not shown) accentuated the difference of insulinemia increase between HMH2 and HFHS (*p* < 0.01) in addition to highlighting the significant increase of insulinemia (*p* < 0.05) of HMH5 compared to the HFHS diet. Therefore, it appeared that the HMH2 product would increase the hyperinsulinemia caused by HFHS, and its derivative product (HMH5) would have the same effect at a lower level. The two supplements necessarily contain common bioactive peptides due to their similar origin, but the treatment that differentiates them will possibly eliminate, from HMH5, some of the peptides responsible for this hyperinsulinemia effect. The previous study did not show such results for HMH2, which would be explained by the lower doses given to the mice [17]. Interestingly, a recent study showed that replacing 70% dietary casein protein with herring milt hydrolysate (dose not specified) in comparison to a high-fat diet reduced *in vivo* obesity, fasting glycemia, and fasting serum insulin, and improved glucose tolerance [28]. The HMH supplements in the present study did not show such positive results. It is therefore possible that the separation that generated the commercial products (HMH1, HMH2, and HMH3) and the EDUF treatment that generated the fractions (HMH4 and HMH5) had concentrated inhibitory peptides in certain supplements and left certain other supplements without bioactive peptides. It is also possible that the offered doses should be increased even further.

In addition, when only HMH1 (original) and HMH4 (fraction) were compared to the HFHS control group (results not shown), HMH1 (original) presented a statistically higher (*p* < 0.05) decrease in glycemia variation than the HFHS diet alone. When only HMH2 (original) and HMH5 (fraction) were compared to the HFHS control group and Chow group (results not shown), HMH5 supplement (fraction) also displayed a statistically higher (*p* < 0.05) decrease than the HFHS diet. Both significant observations occurred 30 min after starting the OGTT. Consequently, it is possible that HMH1 and HMH5 share certain bioactive molecules (peptides, nucleic acids, free amino acids, etc.) that improve glucose intolerance in HFHS-fed mice. Besides, amongst free amino acids and all the peptides in HMH2, the more complex product, some similar free amino acids and peptides are found as well in HMH1 and HMH5 due to the EDUF process, but in different concentrations. In addition, peptides in HMH2 might not have a positive effect on the glucose tolerance due to its complex composition. In fact, bioactive peptides and free amino acids are in lower concentrations or/and would have a lower concentration of inhibitory peptides in HMH1 and HMH5 after the process. Indeed, the HMH5 supplementation contains a lesser concentration of peptides than the HMH2, with 45.26% vs. 48.28%, respectively, since it was submitted to an EDUF run where some peptides have migrated [16]. The HMH1 supplementation contained significantly more peptides than the other two supplements with a concentration of 93.79% [18]. However, the HMH1 peptides had a lower molecular weight than those of the HMH2 and HMH5 supplements (62% of HMH1 peptides had a molecular weight higher than 10,000 Da, while all HMH1 peptides were smaller than 1000 Da), since the HMH1 product was the permeate and the HMH2 product was the retentate [16,19]. Also, the previous studies from Durand et al. [16,17,18] showed that these three supplements had the same free amino acids, but in different concentrations. For example, HMH5 contains more free arginine than HMH2, but less than HMH1, an amino acid which, according to Fu et al. [29], increases glucose uptake *in vitro*. Moreover, according to Durand et al. [18], HMH1 contained peptides and amino acids, but no lipids as found in HMH2 and HMH5. So, even though they share certain peptides and free amino acids, the composition of these three supplements is sufficiently different to justify that HMH2 would not have the same effects as HMH1 and HMH5. Thus, among the results of the studies by Durand et al. [16,17,18], HMH2 shows *in vitro* anti-inflammatory capacity that is not in HMH1 and HMH5. The antioxidant activity of HMH1 (460.67 ± 41.50 μmol TE/g) and HMH5 (279.19 ± 8.67 μmol TE/g) is also greater than the HMH2 (218.32 ± 8.91 μmol TE/g).

### 3.4. Gut Microbiota Analysis

**Figure 5:**
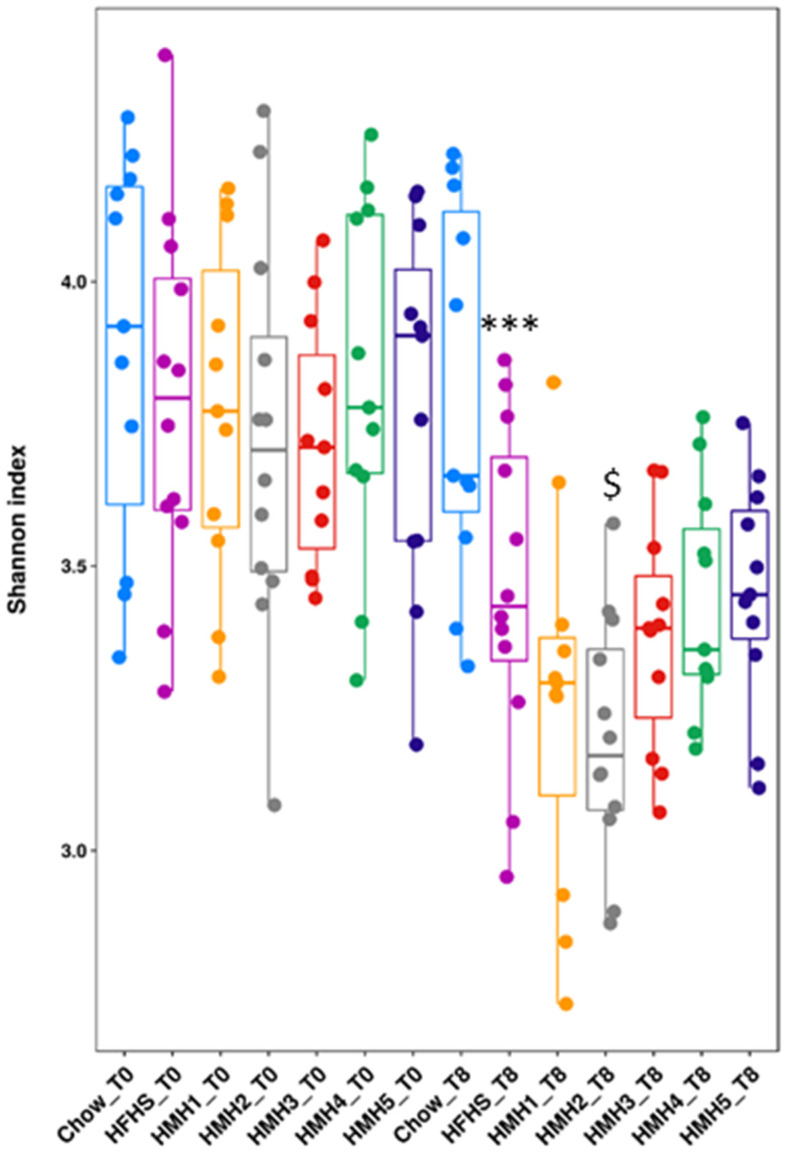
Shannon diversity index calculated at week 0 and 8 for all groups. *** *p* < 0.05 Chow vs. HFHS, $ *p* < 0.05 HFHS vs. HMH2.

In order to assess the impact of the supplementations on gut microbiota composition, fresh feces were collected at the beginning and at the end of the *in vivo* study (week 8). We observed that all mice fed a HFHS diet showed a decreased Shannon index (between week 0 and week 8) (Figure 5) means a decrease of diversity over time, whereas the Chow diet had no impact on the diversity of the gut microbiota. The Shannon index was significantly reduced in the HFHS and the HMH2 groups and to a lesser extent, in the other HMHs-supplemented groups. Similar results were observed in previous studies by Durand et al. [17]. Interestingly, the groups (HFHS, HMHs) that display a low Shannon index value, which represents a low diversity of gut microbial species, also showed obesity with the same severity, consistent with the results of Ley et al. [30] and Le Chatelier et al. [31]. HMH1 and HMH2 showed the lowest unfavorable index values in comparison with HFHS, whereas highest values of Shannon index were observed for the HMH4 and HMH5 fractions (EDUF treatment). This could be explained by the absence of inhibitory peptides in the HMH4 and HMH5 fractions, which were present in the commercial products. Also, the separation that produced HMH1 and HMH2 may be more concentrated in inhibitors in the retentate, HMH2, which would explain how it has the lowest Shannon index. Low diversity is also linked with inflammatory bowel disease according to Qin et al. [32]; an analysis of intestinal inflammation would therefore be relevant in future studies.

**Figure 6:**
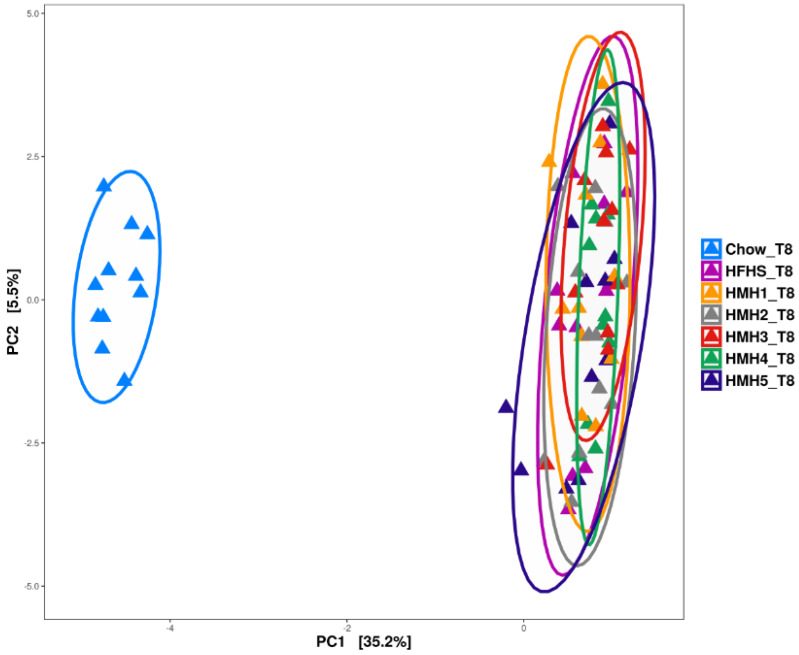
Principal component analysis of fecal samples collected at week 8.

As expected, PCA (Figure 6) showed a clear separation between the Chow and HFHS groups after 8 weeks of treatment. The microbiota of HFHS and HMHs’ supplementation groups largely overlapped, indicating gut bacterial composition was similar between HFHS and HMH groups. Similar results were also observed by Durand et al. [17].

**Figure 7:**
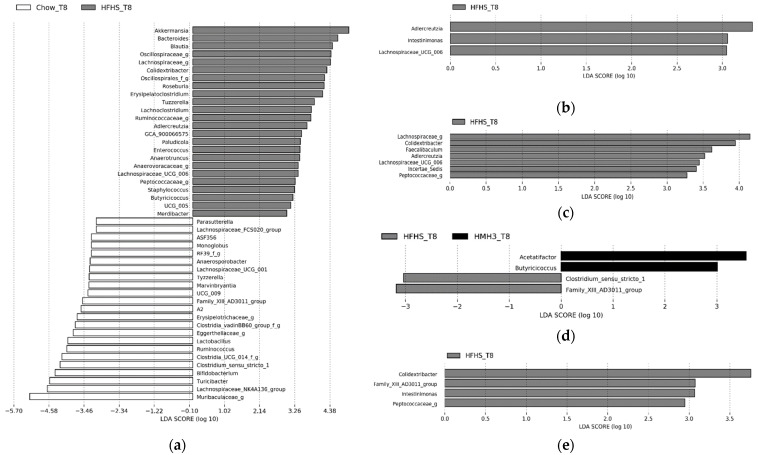
LDA scores computed for differentially abundant bacteria (at the genus level) between (**a**) Chow and HFHS, (**b**) HFHS and HMH1, (**c**) HFHS and HMH2, (**d**) HFHS and HMH3, (**e**) HFHS and MHM5. The labels ‘f’ and ‘g’ indicate unidentified families ad genera, respectively.

**Figure 8:**
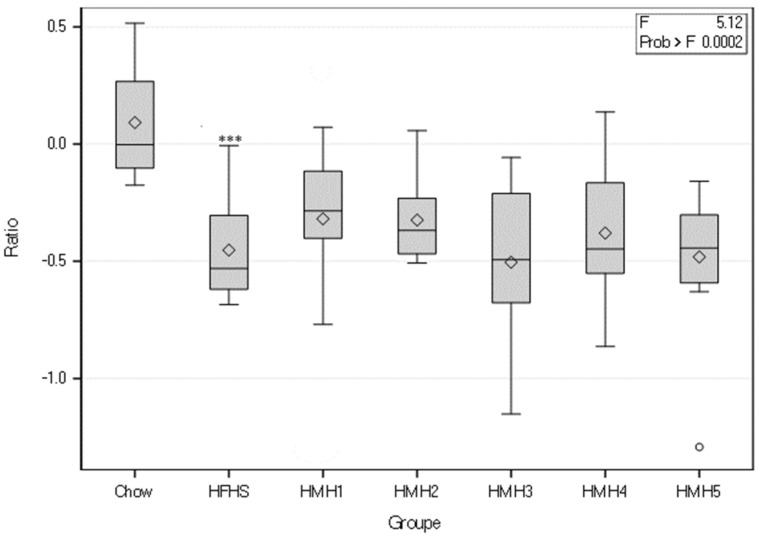
Differential abundance *Bacteroidetes/Firmicutes* ratio calculated for each group. *** *p* < 0.05 Chow vs. HFHS.

The LEfSe analysis showed that in the HFHS group the abundance of several genus, such as *Lachnospiraceae UCG 006*, *Roseburia*, *Colidextribacter*, increased in comparison with the group fed with Chow diet (Figure 7a). Also, in the HFHS group the abundances of genus like *Lactobacillus* decreased (Figure 7a). The Chow and HFHS diets therefore differentiated these bacterial genera, which all belong to the phylum *Firmicutes*. Also, the *Bacteroidetes/Firmicutes* ratio was higher for the Chow diet than the HFHS diet (Figure 8). HMH1 decreased the abundance of *Adlercreutzia* and *Lachnospiraceae UCG 006* compared to HFHS (Figure 7b). At the phylum level, the HMH1 group showed an increase of the *Bacteroidetes/Firmicutes* ratio in comparison with the HFHS group (Figure 8). The abundance of genera *Adlercreutzia*, *Lachnospiraceae UCG006* and *Colidextribacter* were decreased in HMH2 (Figure 7c). HMH3 showed an increase in *Acetatifactor* from the *Lachnospiraceae* family compared to HFHS (Figure 7d). Finally, if the HMH4 treatment has shown no differential abundance, the HMH5 treatment exhibited a decrease of the abundance of *Colidextribacter* compared to the HFHS treatment (Figure 7e).

The HFHS diet increased the abundance of several bacterial genera belonging to *Firmicutes*, a bacterial phylum which in large proportions is often found in the feces of obese hosts, whether mice or humans [33,34]. *GCA 900066575* and *Anaerotruncus* are examples of *Firmicutes,* so their abundances increased in this study, as also reported in the previous *in vivo* study by Durand et al. [17]. Several of these genera, which have been positively correlated with the HFHS diet, belong to the *Lachnospiraceae*. The negative correlation that has been shown between the *Lachnospiraceae* family and animal protein intake by Di lorio’s et al. [35] could also explain how HMH1 or HMH2 supplements may limit the expansion of *Lachnospiraceae UCG 006* despite an HFHS treatment. Moreover, the increase in the *Lachnospiraceae* family has been linked in *in vivo* studies and humans’ studies to metabolic diseases and therefore certain factors of type 2 diabetes [32,36,37,38]. These changes in the abundance of genera belonging to this family could therefore be another indicator of the development of diabetes, which would be confirmed in the HFHS group and still mitigated for the HMH1 and HMH2 groups. However, the HMH3, HMH4, and HMH5 supplements contained similar amounts of animal protein/peptides as HMH1 and HMH2, and therefore should have a similar effect (Table 1). The inhibition of *Lachnospiraceae* production could be caused by peptides present in HMH1 and HMH2, but which would have been removed by EDUF from the HMH4 and HMH5 fractions. For HMH3, the impact of astaxanthin on the stimulation of the development of *Lachnospiraceae* could be proposed to explain a stimulation of the production of *Lachnospiraceae* since HMH2 and HMH3 represent the same product, with the difference that HMH3 contains astaxanthin. However, this effect of astaxanthin has yet to be demonstrated.

According to Riva et al. [39], the abundance of *Bacteroidetes* phylum would be impoverished in obese subjects. The addition of HMH1 supplement to the HFHS diet, however, favored an increase of this phylum even if the development of obesity in this group, according to the weight and the different fatty tissues, was similar to the HFHS group. Interestingly, Larsen et al. [40] showed that a *Bacteroidetes*/*Firmicutes* ratio was greatly increased in subjects with type 2 diabetes compared to healthy subjects. However, Figure 8 shows that none of the HMHs’ supplementation, compared to the HFHS diet, significantly increased this ratio. 

The phylum *Actinobacteria* was identified in the microbiota of obese people by Turnbaugh et al. [41], which supports the increase of one of these genera by the HFHS diet. HMH1 et HMH2 could possibly modulate the microbiota typically associated with an obese individual without lowering body weight since it limited the increase in *Adlercreutzia* belonging to this phylum. This genus is positively correlated with body mass index (BMI) in human and shown in high-fat treatment in mice [42,43]. It is possible that astaxanthin could favor the development of the phylum *Actinobacteria* since the supplement that contains astaxanthin, HMH3, increased the phylum *Actinobacteria*’s abundance compared to the HFHS group. Moreover, this increase is not observable in the group supplemented with HMH2, which is the same supplement as HMH3 without astaxanthin. Although some *in vivo* studies have looked at the effect of astaxanthin on certain genera of *Actinobacteria*, such as *Bifidobacteria*, to date no work has yet shown that astaxanthin has a pre-biotic effect on this phylum or many of its genres [44].

Finally, the HMH5 supplement gave similar results to HMH2 from which HMH5 derives, for example by lowering the abundance of *Colidextricater* and *Peptococcacese*. HMH1, HMH2, HMH3, and HMH5 treatments slightly modulated the gut microbiota in comparison with the HFHS treatment. Finally, it should be determined whether the changes discussed are causes or consequences of obesity and type 2 diabetes.

## 4. Conclusions

This research aimed to evaluate HMHs’ supplementation provided to a HFHS in mice for their possible beneficial effects on the development of obesity and type 2 diabetes associated with the metabolic syndrome. The various supplements did not modulate the increase in weight gain induced by the HFHS diet. In the course of the ITT, a decrease in glycemic variation was observed for HMH2 and HMH5 supplementations. However, in the course of the OGTT, HMH2 increased insulin production. Also, HMH1, HMH2, and HMH5 supplements generated changes in the gut microbiota that are potentially beneficial to the health. Such results could be explained by the peptide composition as well as, in a lower extent, by the presence of PUFA (for HMH2 and HMH5) in the different herring milt hydrolysate supplements (HMH1, HMH2, and HMH5), which can improve metabolic health on several levels. Furthermore, the difference in activity between HMH2 and its HMH5 fraction could be explained by the presence of inhibitory peptides in HMH2 or by a concentration of bioactive peptides in HMH5. Nevertheless, more studies will be required to verify their transposition in human models. Clinical results concluding with a limited reduction in the risk factors of type 2 diabetes by the supplements HMH1, HMH2, and HMH5 would represent a new therapeutic avenue for the prevention of this chronic disease.

## Figures and Tables

**Table 1:** Chemical composition of the different herring milt hydrolysates (HMH).

	HMH1	HMH2	HMH3	HMH4	HMH5
Protein/peptide (%)	93.8 *	48.3 *	47.0 *	93.1	74.4
Lipids (%)	-	18.5 *	26.0 *	-	-
Nucleic acid (%)	7.3 *	27.3 *	7.0 *	-	-
Astaxanthin (ppm)	-	-	500 *	-	-

* Values from a previous study [17]. - Not available.

**Table 2:** Effects of HMHs’ supplementation on mice body characteristics.

	Chow	HFHS	HMH1	HMH2	HMH3	HMH4	HMH5
Total weight gain (g)	3.38 ± 0.64 ***	8.47 ± 2.29	9.79 ± 2.47	9.42 ± 2.14	9.65 ± 3.48	9.72 ± 2.28	9.98 ± 2.91
Total energy intake (kcal)	518.16 ± 4.76 ***	626.87 ± 14.14	621.97 ± 14.62	629.13 ± 8.32	667.97 ± 16.66	660.80 ± 20.06	639.22 ± 14.03
Visceral fat pad (g)	0.88 ± 0.18 ***	2.58 ± 0.85	2.92 ± 1.14	2.85 ± 0.65	2.81 ± 1.17	2.85 ± 0.89	2.90 ± 0.99
Subcutaneous fat pad (g)	0.27 ± 0.04 ***	0.61 ± 0.21	0.69 ± 0.24	0.67 ± 0.14	0.70 ± 0.31	0.68 ± 0.20	0.74 ± 0.26
Brown adipose tissue (g)	0.07 ± 0.01 **	0.10 ± 0.03	0.10 ± 0.03	0.10 ± 0.02	0.11 ± 0.03	0.10 ± 0.02	0.10 ± 0.02
Total lean mass (g)	18.9 ± 1.0	19.6 ± 1.3	20.0 ± 1.6	19.9 ± 1.2	20.2 ± 2.1	20.9 ± 1.3	19.9 ± 1.1
Total fat mass (g)	2.3 ± 0.9 ***	6.2 ± 2.1	6.6 ± 2.5	7.1 ± 2.0	6.4 ± 3.1	6.9 ± 2.2	6.4 ± 2.3
Gastroc (2) (g)	0.25 ± 0.02	0.26 ± 0.02	0.26 ± 0.02	0.26 ± 0.01	0.27 ± 0.02	0.27 ± 0.02	0.26 ± 0.02
Soleus (2) (g)	0.01 ± 0.00	0.02 ± 0.00	0.02 ± 0.00	0.02 ± 0.00	0.02 ± 0.00	0.02 ± 0.00	0.02 ± 0.00
Brain (g)	0.44 ± 0.01	0.42 ± 0.02	0.44 ± 0.01 @	0.44 ± 0.01	0.44 ± 0.02	0.44 ± 0.01	0.42 ± 0.02
Heart (g)	0.12 ± 0.01	0.13 ± 0.01	0.14 ± 0.01	0.13 ± 0.01	0.14 ± 0.02	0.13 ± 0.01	0.13 ± 0.01
Kidneys (2) (g)	0.32 ± 0.03	0.33 ± 0.03	0.36 ± 0.06	0.35 ± 0.03	0.35 ± 0.05	0.35 ± 0.04	0.33 ± 0.03
Liver (g)	1.05 ± 0.07	1.08 ± 0.13	1.01 ± 0.20	1.05 ± 0.13	1.03 ± 0.16	1.06 ± 0.14	1.07 ± 0.14
Pancreas (g)	0.26 ± 0.01	0.28 ± 0.02	0.31 ± 0.07	0.29 ± 0.04	0.31 ± 0.07	0.29 ± 0.05	0.27 ± 0.02

** *p* < 0.01 vs. HFHS, *** *p* < 0.001 vs. HFHS, @ *p* < 0.05 vs. HFHS

## Data Availability

Data is contained within the article.
